# Solid Lipid Nanoparticles of Albendazole for Enhancing Cellular Uptake and Cytotoxicity against U-87 MG Glioma Cell Lines

**DOI:** 10.3390/molecules22112040

**Published:** 2017-11-22

**Authors:** Gregory Marslin, Karthik Siram, Xiang Liu, Vinoth Kumar Megraj Khandelwal, Xiaolei Shen, Xiang Wang, Gregory Franklin

**Affiliations:** 1 Chinese-German Joint Laboratory for Natural Product Research, Qinling-Bashan Mountains Bioresources Comprehensive Development C.I.C., College of Biological Science and Engineering, Shaanxi University of Technology, Hanzhong 723000, China; liuxiang8885@163.com (X.L.); 17809268805@163.com (X.S.); 15029541697@163.com (X.W.); 2 Department of Pharmaceutics, PSG College of Pharmacy, Coimbatore, Tamilnadu, 641004, India; karthiksiram@gmail.com; 3 Department of Translational Pharmacology, Consorzio Mario Negri Sud, Santa Maria Imbaro 66030, Italy; drvinothk@gmail.com; 4 Department of Integrative Plant Biology, Institute of Plant Genetics of the Polish Academy of Sciences, Strzeszyńska 34, 60-479 Poznań, Wielkopolska, Poland

**Keywords:** albendazole, solid lipid nanoparticles, U-87 MG cells, cytotoxicity, cellular uptake

## Abstract

Albendazole (ABZ) is an antihelminthic drug used for the treatment of several parasitic infestations. In addition to this, there are reports on the anticancer activity of ABZ against a wide range of cancer types. However, its effect on glioma has not yet been reported. In the present study, cytotoxicity of ABZ and ABZ loaded solid lipid nanoparticles (ASLNs) was tested in human glioma/astrocytoma cell line (U-87 MG). Using glyceryl trimyristate as lipid carrier and tween 80 as surfactant spherical ASLNs with an average size of 218.4 ± 5.1 nm were prepared by a combination of high shear homogenization and probe sonication methods. A biphasic in vitro release pattern of ABZ from ASLNs was observed, where 82% of ABZ was released in 24 h. In vitro cell line studies have shown that ABZ in the form of ASLNs was more cytotoxic (IC50 = 4.90 µg/mL) to U-87 MG cells compared to ABZ in the free form (IC_50_ = 13.30 µg/mL) due to the efficient uptake of the former by these cells.

## 1. Introduction

Development of new drugs with increased activity against cancer cells is the major focus of the pharmaceutical industry. Unfortunately, many of the new drugs are often toxic to the healthy cells as well. Hence, there is a space for the development of drugs that are toxic only to cancer cells. Although albendazole (ABZ) is known for its antihelminthic activity, recent studies show that it possesses significant anticancer activity as well. ABZ is an affordable drug, which exhibits antihelminthic activity even at a very low concentration (7.5 mg/kg) [1]. The ability of ABZ to destroy the β tubulin structures of the helminthes has also been exploited to kill tumor cells. Activities of ABZ against colorectal cancer and peritoneal carcinomatosis have been demonstrated respectively using HT-29 cell line and xenograft models [2]. Later, the ability of ABZ to inhibit cell proliferation, vascular endothelial growth factor and tumor growth was discovered [3]. In addition, ABZ could also arrest the mitotic phase and induce apoptosis in human gastric cancer cells [4]. Unlike the other anticancer drugs, ABZ is remarkably safe at high doses (600 mg) even after multiple administrations [5].

In spite of the non-toxicity of ABZ to healthy cells, due to poor gastrointestinal absorption (5%) and low bioavailability, often multiple doses of ABZ need to be administered to improve the therapeutic efficiency. To improve the solubility as well as bioavailability of this drug, several formulation approaches such as particle size reduction, complexation, emulsion, and suspension have been investigated [6,7]. In addition, development of novel nanoformulations has also been approached as evidenced by the following reports. Chitosan and tripolyphosphate nanoparticles containing ABZ were prepared to enhance solubility and cytotoxicity [8]. Delivery of ABZ as lipid nanocapsules enhanced the oral bioavailability and efficacy of cystic echinococcosis therapy in experimentally infected mice [9]. Similarly, ABZ loaded solid lipid nanoparticles (SLNs) were tested for the treatment of *Toxocara canis* infection in vivo [10]. Further, the nanoencapsulation of ABZ in chitosan-coated PLGA nanoparticles could enhance the anticancer activity [11].

Nowadays, chemotherapeutic agents can be delivered directly into the cancer cells, when encapsulated in a nanocarrier [12,13]. Among the various nanocarriers available, SLNs have gained much attention, as they can provide the advantages of colloidal carriers like liposomes and emulsions. SLNs are colloidal carriers with a solid lipid matrix that can solubilize lipophilic molecules and usually stabilized by surfactants [14,15]. Tumor cells could efficiently uptake SLNs and thereby systemic toxicity of the drug could be minimized [16]. All the more, they are biodegradable, nontoxic and also economically viable [17,18,19,20].

The incidence and challenges posed by glioma and the aforementioned advantages of SLNs motivated us to test the cytotoxicity of ABZ both in the free form as well as encapsulated form (ASLNs) in U-87 MG human glioblastoma cells in the present study. Results presented here reveal that the delivery of ABZ in the form of SLNs was cytotoxic to U-87 MG cells due to their efficient uptake by these cells.

## 2. Results and Discussion

Nanoformulations are gaining significant attention in recent years due to their ability to achieve site-specific action of anticancer drugs at a therapeutically optimal rate [21,22]. Delivery of anticancer drugs in an encapsulated form not only protects the drug against chemical and enzymatic degradation, but also reduces the unintended toxicity towards normal cells. Moreover, development of affordable cancer therapies with reduced toxicity is essential to increase patient compliance [23]. The affordability, safety and non-toxicity of ABZ prompted us to develop ASLNs and to study their uptake and cytotoxicity in U-87 MG glioma cell line for the first time. 

**Figure 1:**
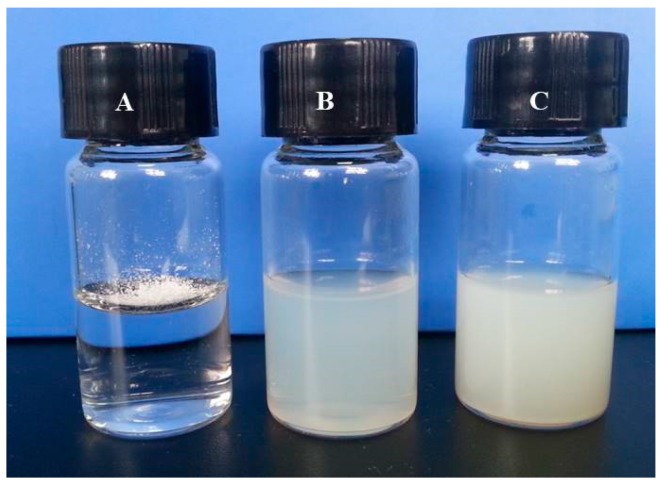
Photographs showing water solubility of (**A**) Albendazole (ABZ); (**B**) Blank solid lipid nanoparticles (BSLNs) and (**C**) Albendazole loaded solid lipid nanoparticles (ASLNs).

ABZ (2 mg) added to 5 mL of water did not disperse uniformly due to its poor solubility (Figure 1A). However, we could generate uniform colloidal dispersion of BSLNs (Figure 1B) and ASLNs (Figure 1C) using high shear homogenization and probe sonication. 

**Figure 2:**
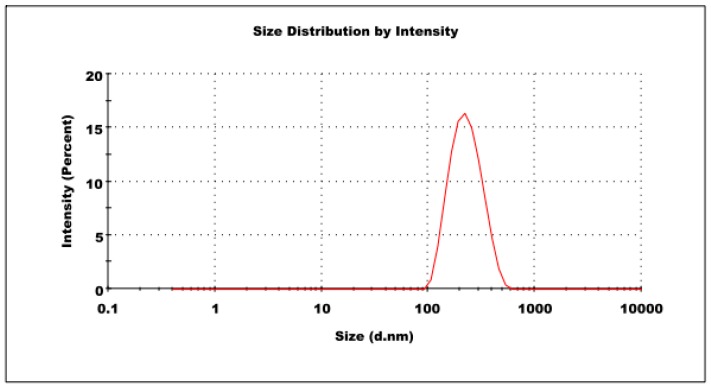
Particle size distribution of ASLNs.

**Figure 3:**
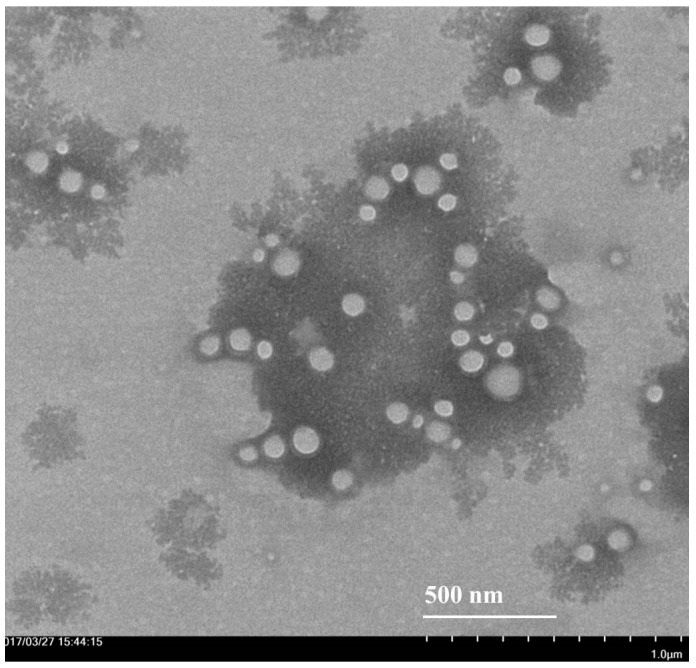
Transmission electron microscopic (TEM) image of ASLNs.

ASLNs prepared in the present study possessed an average size of 218.4 ± 5.1 nm in diameter (Figure 2). The low PDI value (0.16 ± 0.04) obtained for the formulation indicates that the ASLNs are homogeneous. The mild negative zeta potential (−12.4 ± 1.32 mV) of the ASLNs might be attributed to partial hydrolysis of the ester groups of glyceryl trimyrsistate at higher temperatures during the preparation. The homogeneity, spherical shape and lack of aggregation were further confirmed by transmission electron microscope (TEM) images of ASLNs (Figure 3). The whitish spheres are the ASLNs, whereas the dark spot seen around them is due to sodium phosphotungstate solution (1%, *w*/*w*) used for the negative staining of ASLNs [24].

Encapsulation efficiency provides an estimation of the amount of drug encapsulated within the nanoparticles. The preparation method used in the present study resulted in the encapsulation of 62 ± 4.2% ABZ within the SLNs. Better encapsulation efficiency (81%) of ABZ was achieved in PEG-ylated liposomes [25]. Hence, the variation in the encapsulation efficiency obtained in the present and previous studies could be attributed to the PEGylation [26].

**Figure 4:**
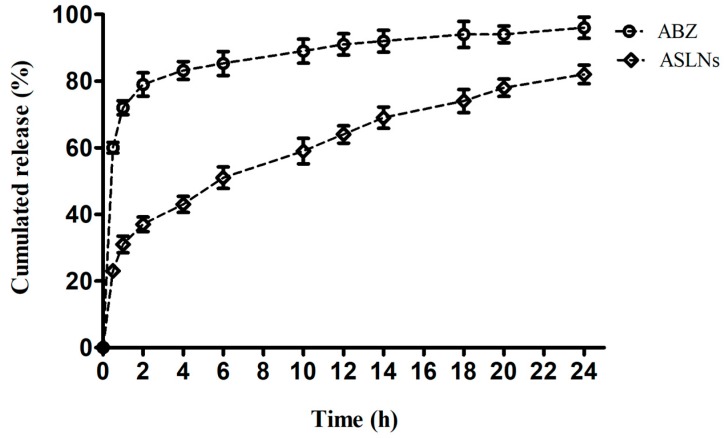
In vitro drug release profile of ASLNs in PBS (pH 7.4) containing 1% tween 80.

Figure 4 shows the in vitro release patterns of ABZ from ASLNs and free ABZ from the dialysis bag to the surrounding PBS. An initial burst release for about 2 h, followed by the steady release of ABZ from ASLNs (for 24 h) depicts a biphasic release profile of entrapped ABZ. Although about 31% of the drug was released from ASLNs within 2 h, a slow release was evidenced thereon reaching only 82% in 24 h. On the other hand, about 72% and 96% of free ABZ from the dialysis bag reached the medium respectively in 2 and 24 h. While the initial burst release might be due to the free drug adhered to the surface of ASLNs, the later sustained release could be attributed to the slow release of ABZ encapsulated within the ASLNs. Similar biphasic release pattern was observed in ABZ loaded within Compritol 888 ATO SLNs [10].

**Figure 5:**
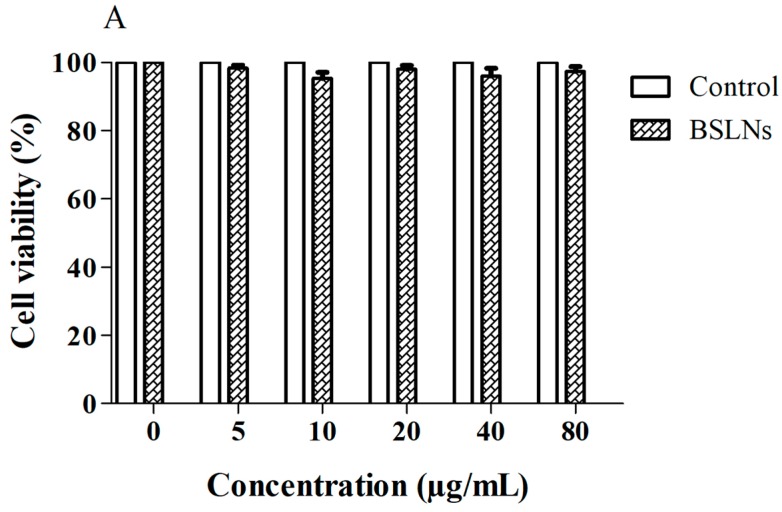

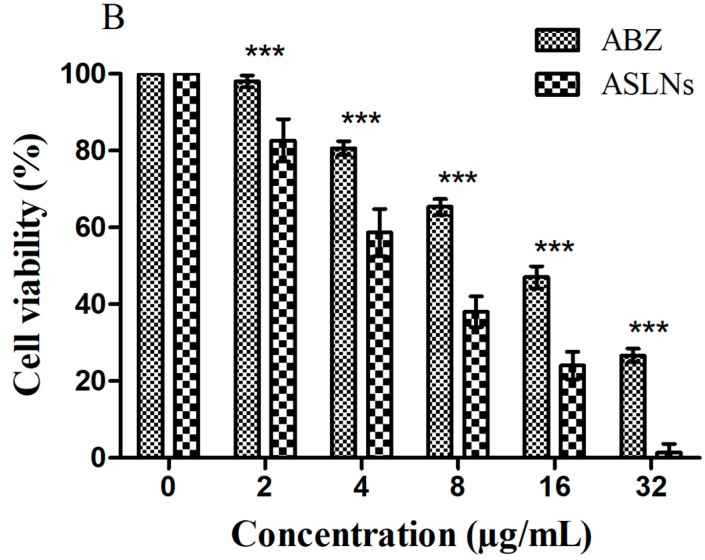
Graph showing the cytotoxic potential of control and BSLNs against U-87 MG cells (mean ± SEM, *n* = 3). (**A**) Comparison of cytotoxicity of ABZ and ASLNs in U-87 MG cells (mean ± SEM, *n* = 3). (**B**) Statistical analysis of data was performed using 2-way ANOVA, followed by Bonferroni’s Multiple Comparison Test. *** Denotes significance at *p* < 0.001.

MTT based cell proliferation assay with U-87 MG cells showed that ASLNs were more cytotoxic (IC50: 4.90 µg/mL) than ABZ (IC50: 13.30 µg/mL). Since the BSLNs did not show any cytotoxicity to these cells, we can safely conclude that the cytotoxicity of ASLNs is due to the effect of encapsulated ABZ and not due to the components used in the preparation of SLNs such as glyceryl trimyristate and tween 80 (Figure 5A). Significantly higher inhibition of the growth of U-87 MG cells was noticed in ASLNs compared to its equivalent ABZ (Figure 5B), suggesting that the enhanced cytotoxicity of ASLNs is due to the SLN formulation.

**Figure 6:**
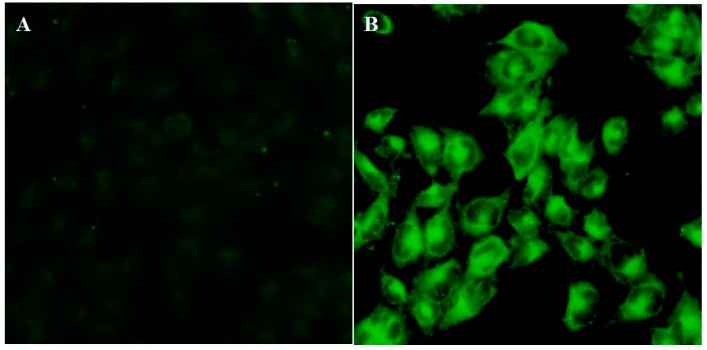
Fluorescence imaging of U-87 MG cells after 2 h of incubation. (**A**) Cells incubated with curcumin not showing any fluorescence; (**B**) Cells incubated with CSLNs showing an intense green fluorescence revealing the efficient uptake of CSLNs by these cells.

In order to check whether the increased cytotoxicity of ASLNs is due to higher uptake of ABZ into the cells in the form of nanoparticles, we performed a cellular uptake study using CSLNs and curcumin due to their fluorescence characteristics [27]. Figure 6 represents the images of U-87 MG cells visualized under a fluorescence microscope after incubating them with curcumin and CSLNs. As seen in the images, CSLNs treated cells fluoresced intensively, whereas the cells treated with curcumin did not fluoresce, indicating that CSLNs were efficiently taken up by the U-87 MG cells possibly via endocytosis [28]. These results clearly show that the enhanced cytotoxicity of ASLNs observed in U-87 MG cells compared to ABZ in the MTT assay is due to the efficient uptake of ASLNs.

## 3. Methods and Materials

### 3.1. Chemicals and Reagents

ABZ was obtained as a gift sample from Vetindia Pharmaceuticals Limited (Hyderabad, Andhra Pradesh, India). Curcumin, phosphate buffer saline (PBS, pH 7.4) and glyceryl trimyristate were purchased from Solarbio Life Sciences (Beijing, China). Tween 80 and all solvents used in this study were acquired from Tianjin Chemical and Reagents (Tianjin, China).

### 3.2. Preparation of SLNs

ASLNs were prepared by a combination of high-speed homogenization and ultrasonication method. Briefly, 500 mg of glyceryl trimyristate was heated to 5 °C above its melting point to form a clear lipid phase and 10 mg of ABZ was added in the melted lipid to form a clear lipid phase. Similar to that of the lipid phase, the aqueous phase containing 25 mL of water and 1% of tween 80 was also heated to the same temperature. Then, the lipid phase was added into the aqueous phase to form an emulsion. This emulsion was homogenised using a high-speed homogenizer (ANGNI Instruments, AD300L-H, Ningbo, China) at 15,000 RPM for 5 min and sonicated for 3 min (Ningbo Scientz Biotechnology Co Ltd., Ningbo, China) at 50% amplitude. Finally, the hot oil-in-water nanoemulsion was cooled down to room temperature to form ASLNs. In the same manner, curcumin loaded SLNs (CSLNs) were prepared, but instead of ABZ, 10 mg of curcumin was used to prepare the lipid phase. SLNs prepared without any drug served as BSLNs.

### 3.3. Evaluation of Particle Size and Polydispersity Index (PDI) 

To determine the average particle size and PDI of ASLNs, photon correlation spectroscopy was performed in a Malvern Zetasizer (Nano ZS90, Malvern Instruments, Malvern, UK) at 25 °C. ASLNs were loaded in a polystyrene cuvette and the measurements were performed in triplicate.

### 3.4. Zeta Potential

The zeta potential of the SLNs was measured by Laser Doppler velocimetry using Malvern Zetasizer at 25 °C. The sample was diluted in double distilled water and a zeta dip cell was used to measure the zeta potential.

### 3.5. Morphological Characterization

The morphology of the ASLNs was examined by transmission electron microscopy (TEM). Approximately 10 µL of ASLNs was placed on a copper grid and allowed to air dry. The air-dried sample was negatively stained with sodium phosphotungstate solution (1%, *w*/*v*) and images were taken in a transmission electron microscope (Hitachi-HT7700, Hitachinaka, Japan).

### 3.6. Entrapment Efficiency of ASLNs

The encapsulation efficiency was estimated by an ultracentrifugation method. Briefly, 1 mL of ASLNs was centrifuged at 20,000 rpm (21,242× *g*) for 45 min at 4 °C. The supernatant was collected and the amount of ABZ (which corresponds to the free drug) was measured at 265 nm in UV spectrophotometer (Mapada UV-6100 S, Shanghai Mapada Instruments Co Ltd., Shanghai, China) [25]. The sensitivity of the developed method was found to be in the range of 2–20 mcg/mL (r^2^ value = 0.09997). Supernatant obtained from BSLNs in the same manner was used as blank. The entrapment efficiency was calculated from the following equation.

$$\mathrm{Encapsulation}\;\mathrm{efficiency}\;\left(\%\right)=\frac{\mathrm{Total}\;\mathrm{drug}-\mathrm{Free}\;\mathrm{drug}}{\mathrm{Total}\;\mathrm{amount}\;\mathrm{used}\;\mathrm{for}\;\mathrm{preparation}}\times 100$$

### 3.7. In Vitro Drug Release

ASLNs (5 mL) was placed in a dialysis bag (M.W: 12,000–14,000 Daltons), hermetically sealed and immersed into a beaker containing 50 mL of PBS (pH 7.4) and 1% tween-80 [29]. The buffer solution was maintained at 37 ± 1 °C under continuous magnetic stirring (50 rpm). At predetermined time intervals (0–24 h), 1 mL buffer from the beaker was withdrawn and replaced by equal amount of fresh PBS. The amount of ABZ in the samples was determined by UV spectroscopy at 265 nm.

### 3.8. In Vitro Cellular Uptake 

The uptake of ASLNs by U-87 MG cells (human glioblastoma–astrocytoma, epithelial-like cell line) was analyzed indirectly using CSLNs exploiting the fluorescence characteristics of curcumin. Cells seeded in 24-well plates at a concentration of 0.05 × 10^6^ cells/well were maintained at 37 °C and 5% CO_2_ in a cell culture chamber for cell adhesion. After 24 h, the medium was replaced with fresh medium containing either CSLN or free curcumin and incubated at the same conditions [27]. After 2 h, the wells were washed with ice-cold PBS and the cells were analyzed under a fluorescence microscope (Olympus 1X71, Olympus, Tokyo, Japan).

### 3.9. Cytotoxicity of Nanoparticles

The cytotoxic effect of ABZ and ASLNs was determined in U-87 MG cells via MTT assay. Cells were plated in flat bottom 96-well plates at a concentration of 5000 cells/well and maintained at 37 °C and 5% CO_2_ in a cell culture chamber for 24 h. Then the culture medium was replaced with 200 µL of fresh medium containing different concentrations of ABZ (5, 10, 20, 50, 100 µg/mL) or its equivalent concentration of ASLNs and incubated as described above. To check the cytotoxicity of components other than drug, BSLNs were added at concentrations equivalent to ASLNs in separate wells. After 24 h of incubation, medium from all the wells was replaced with 100 µL of medium containing 500 µg/mL MTT and incubated for another 1 h. Then, 100 µL SDS solution (20% *w*/*v*, water: DMF at 1:1 ratio, pH 4.7) was added to each well and incubated for 24 h to dissolve the purple formazan crystals. Absorbance at 570 nm of the resulting solution was read in an ELISA plate reader (Victor 1420, PerkinElmer, Shelton, CT, USA) to quantify the amount of formazan formed, which is directly proportional to the number of viable cells. The cell viability in the presence of ABZ, ASLNs and BSLNs were compared with untreated control cells.

### 3.10. Statistical Analyses of Data

Statistical analyses were performed using Prism software (Version 5, GraphPad, GraphPad Software Inc., La Jolla, CA, USA). Results are expressed as the mean of three replicates ± standard deviation (SD). Statistical significance between treatments was analyzed by two-way ANOVA followed by Bonferroni’s Multiple Comparison Test.

## 4. Conclusions

The results presented here clearly show that ABZ has cytotoxicity against U-87 MG cells. When delivered as SLNs (ASLNs), the cytotoxic potential of ABZ could be further improved, which can be attributed to the efficient uptake of SLNs by these cells. Although we believe that ASLNs would be more bioavailable compared to ABZ, in vivo studies are required to confirm this, which is underway in our laboratory.
